# Usability Evaluation of a Central Monitoring System with AI-Based Cardiac Arrest Prediction in the ICU

**DOI:** 10.3390/jcm15062261

**Published:** 2026-03-16

**Authors:** Jiyoon Oh, Yourim Kim, Wonseuk Jang

**Affiliations:** 1 Department of Medical Device Engineering and Management, Yonsei University College of Medicine, Seoul 06229, Republic of Korea; 5wldbs27@naver.com (J.O.); yrkim0224@yuhs.ac (Y.K.); 2 Medical Device Usability Research Center, Gangnam Severance Hospital, Yonsei University College of Medicine, Seoul 06230, Republic of Korea

**Keywords:** usability test, summative evaluation, cardiac arrest, prediction, central monitoring systems

## Abstract

**Background/Objectives**: The incidence of cardiac arrest among critically ill patients has been increasing, with many patients experiencing clinical exacerbation prior to the event. Early detection and rapid treatment are essential to reduce the risks associated with cardiac arrest; however, difficulties such as limited ICU resources and inadequate monitoring of vital signs reduce the effectiveness of treatment. Various cardiac arrest prediction systems have been developed to overcome these issues. This study performed a summative evaluation of a Central Monitoring System with AI-based Cardiac Arrest Prediction. **Methods**: A summative usability evaluation was conducted in a simulated ICU environment with 22 ICU nurses experienced in using patient monitoring devices. Participants completed tasks based on the device workflow and then filled out the System Usability Scale (SUS) and satisfaction surveys, with task performance and survey responses analyzed to assess usability. **Results**: The usability test achieved a task success rate of 90%, with critical tasks achieving success rates ranging from 73% to 100%. The SUS score was 67.3 (“OK”), and the satisfaction survey showed an average score of 4.5, indicating generally positive user perception. **Conclusions**: Participants generally rated the system as acceptable, although some tasks showed lower success rates due to design issues such as poor button visibility. Further studies in clinical settings are needed to evaluate the system’s effectiveness, user experience, and contribution to the timely detection of cardiac arrest.

## 1. Introduction

The incidence of cardiac arrest among patients admitted to the intensive care unit (ICU) has been increasing annually, and the mortality rate of patients who experience cardiac arrest is higher than that of those who do not [1,2]. Up to 80% of patients show signs of clinical exacerbation before experiencing cardiac arrest [3,4]. Early detection and rapid treatment are important to prevent further exacerbation following cardiac arrest. However, difficulties such as limited ICU resources, inadequate vital signs monitoring, and untimely corrective actions reduce the effectiveness of treatment [1,5]. To overcome these issues, various countries have investigated strategies such as the operation of Medical Emergency Teams, specialized training programs for managing critically ill patients, and the development of systems to predict patient exacerbation [1,6,7,8].

Several studies have been conducted on the development of cardiac arrest prediction systems for use in emergency departments and ICUs. Using databases such as MIMIC (Medical Information Mart for Intensive Care), eICU-CRD (eICU Collaborative Research Database), researchers have developed prediction algorithms based on patients’ electrocardiogram (ECG) data and vital signs [9,10]. These algorithms have demonstrated high predictive performance, with an AUC of 0.80 or higher, indicating their potential for real-time prediction of cardiac arrest in the ICU [9,10]. Despite the high predictive performance reported in previous studies, the real-world implementation of AI-based clinical decision support systems remains limited due to challenges related to workflow integration, interface usability, and clinician acceptance [11].

In this study, we developed a Central Monitoring System with Cardiac Arrest Prediction for use in ICUs to predict the risk of cardiac arrest in real time. Vital signs measured by the patient monitor are sent to the central monitoring system, and an artificial intelligence-based algorithm analyzes the data to estimate the patient’s risk of cardiac arrest. The estimated risk can be reviewed on the central monitoring system. The system was designed to generate predictions based on routinely collected vital signs and patient information in the ICU without requiring additional tests, enabling continuous and stable application in clinical environments [8].

ICUs are environments designed to provide treatment for critically ill patients and are equipped with various devices such as patient monitors and ventilators [12,13]. In particular, patient monitors are widely used for continuous monitoring, enabling the observation of parameters such as invasive blood pressure, electroencephalogram (EEG), electrocardiogram (ECG), carbon dioxide (CO_2_) levels, and transcutaneous oxygen saturation (SpO_2_) [14,15]. ICU nurses are responsible for providing care to critically ill patients and making decisions in critical situations, resulting in higher workload compared with other departments [12]. The demanding work environment and the quality of medical equipment contribute to ICU nurses’ occupational stress, which can negatively affect patient outcomes [16]. In addition, frequent alarms may lead to alarm fatigue, which can disrupt workflow and compromise patient safety [17]. In such high workload settings, use errors related to medical devices are more likely to occur [14]. Therefore, before implementing new technologies, particularly systems with alarm-based prediction functions, it is important to perform systematic usability testing to identify potential use errors and analyze their root causes [18,19].

A usability test focuses on evaluating whether users can complete tasks that are based on the device’s workflow [19]. Formative evaluation is conducted during the development process to evaluate the suitability of the user interface and to identify unexpected use errors [20]. Summative evaluation is performed to demonstrate that the medical device can be used safely and effectively without serious use errors [20]. While previous studies have mainly focused on the predictive performance of cardiac arrest algorithms, this study performed a summative evaluation of a Central Monitoring System with AI-based Cardiac Arrest Prediction in a simulated environment with 22 ICU nurses.

## 2. Materials and Methods

### 2.1. Central Monitoring System with AI-Based Cardiac Arrest Prediction

#### 2.1.1. Configuration

**Figure 1:**
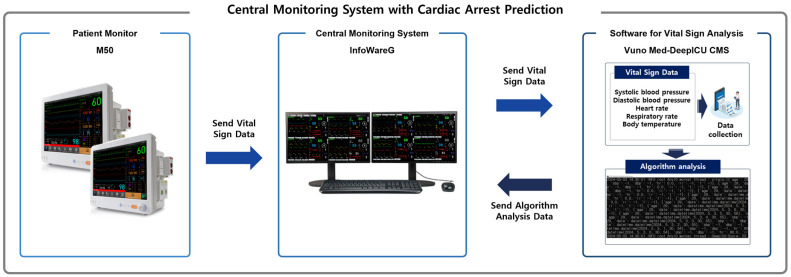
Central Monitoring System with Cardiac Arrest Prediction.

As illustrated in Figure 1, a Central Monitoring System with Cardiac Arrest Prediction consists of a patient monitor, a central monitoring system, and software for vital sign analysis.

The M50 (Mediana Co., Wonju-si, Gangwon-do, Republic of Korea) is a patient monitor (PM) designed to monitor patients by measuring vital signs such as electrocardiogram (ECG), heart rate (HR), non-invasive blood pressure (NIBP), oxygen saturation (SpO_2_), pulse rate (PR), respiration rate (RR), and body temperature.

InfoWareG (Mediana Co., Wonju-si, Gangwon-do, Republic of Korea) is a central monitoring system (CMS) that integrates data from multiple patient monitors and generates visual or auditory alarms when signs of patient exacerbation are detected.

VUNO Med-DeepICU CMS (v1.0.X; VUNO Inc., Seoul, Republic of Korea) is software for vital sign analysis designed to support diagnosis, analysis, and simulated treatment by integrating patients’ vital signs. It collects and analyzes systolic and diastolic blood pressure, pulse rate, respiratory rate, and body temperature to present a cardiac arrest prediction score ranging from 0 to 100. Previous study has demonstrated the high predictive performance of VUNO Med-DeepICU CMS for cardiac arrest prediction [8].

The patient monitor (M50) collects the patient’s vital signs and transmits the data to the central monitoring system (InfoWareG). The central monitoring system then forwards the data to the vital sign analysis software (VUNO Med-DeepICU CMS). The software analyzes the patient’s age, vital signs, and the timing of measurements to generate a cardiac arrest prediction score ranging from 0 to 100. The central monitoring system displays the cardiac arrest prediction score on the screen, enabling real-time monitoring.

#### 2.1.2. GUI (Graphical User Interface)

**Figure 2:**
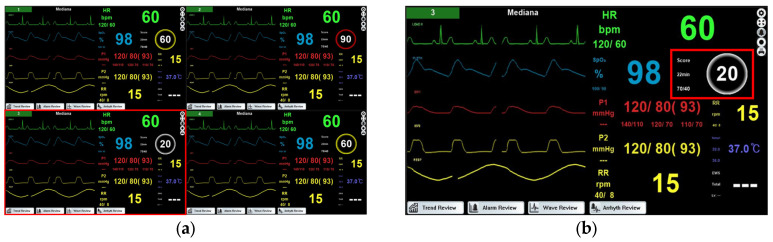
Central monitoring system screens: (**a**) the screen monitoring multiple beds; (**b**) one bed in the screen monitoring multiple beds.

Figure 2a shows the central monitoring system screen displaying multiple beds simultaneously. Figure 2b is an enlarged view of the area highlighted by the red box in Figure 2a, showing the main screen of the central monitoring system for a single bed. The cardiac arrest prediction score, generated by the software for vital sign analysis, is displayed on this screen. The area where the score appears is highlighted with a red box in Figure 2.

**Figure 3:**
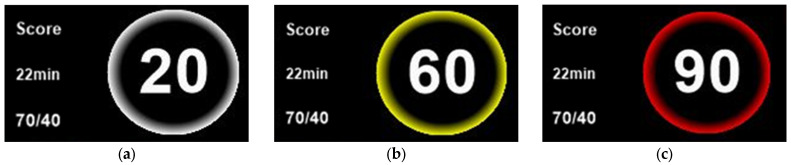
Detailed view of cardiac arrest prediction score section (**a**) normal; (**b**) primary alarm, (**c**) secondary alarm.

The cardiac arrest prediction score is represented as a number ranging from 0 to 100, with higher scores indicating an increased risk of cardiac arrest. By clicking the “score” area on Figure 3, users can view the scores generated over a defined interval. The “22 min” indicates the time since the score was generated, and the score is updated every 30 min. The “70/40” represents the severity step 2 limit (70, secondary alarm) and step 1 limit (40, primary alarm), defining the risk levels of cardiac arrest. According to the ANSI/AAMI HE75:2009 (R2018) standard, a white color is used to convey primary information on a black background, a yellow color indicates a medium or low priority alarm for medical purposes and caution for potential hazards, and a red color signals a high-priority alarm and danger [21]. In the central monitoring system, these colors are applied to the borders of the cardiac arrest prediction score circles to indicate the risk level to the user. A white border represents a score of 0–40, indicating a normal status (Figure 3a). A yellow border represents a score of 41–70, indicating a primary alarm that suggests an increased risk of cardiac arrest (Figure 3b). A red border represents a score of 71–100, indicating a secondary alarm that reflects a high risk of cardiac arrest (Figure 3c).

**Figure 4:**
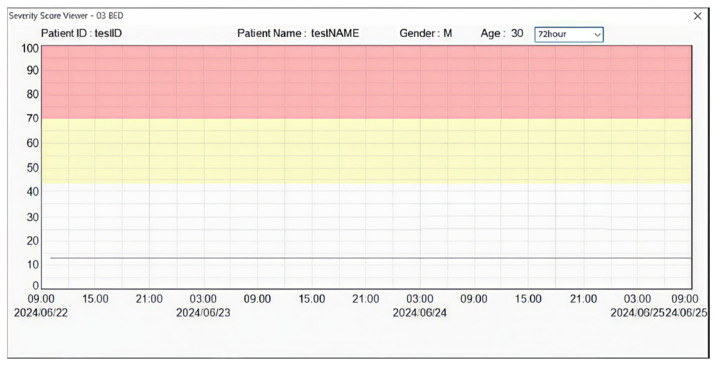
Screen with cardiac arrest prediction scores over 72 h.

Figure 4 shows the screen displaying the cardiac arrest prediction scores generated within a 72-h period. Users can view the generated scores for 24-h or 48-h periods by selecting a period on the screen.

### 2.2. Study Design

In accordance with IEC 62366-1 recommendations, a total of 22 ICU nurses with prior experience using a patient monitor or a central monitoring system were recruited via email, exceeding the minimum of 15 participants required for a summative evaluation. All participants provided informed consent before participating in this study, and their demographic information, including sex, age, work experience, and user experience with similar devices, was recorded. They performed tasks based on predefined use scenarios and, following the usability test, completed the System Usability Scale (SUS) questionnaire and a satisfaction survey.

This study was approved by the Institutional Review Board (IRB) of Gangnam Severance Hospital (Approval No.: 3-2024-0212, approved on 25 July 2024) and conducted in the Medical Device Usability Research Center (Gangnam-gu, Seoul, Republic of Korea) from July 2024 to October 2024.

### 2.3. Study Procedure

One participant was involved per test session. The facilitator introduced the test procedure and obtained informed consent forms from each participant. A training moderator conducted a 15-min training session covering device operation, GUI navigation, and the cardiac arrest prediction score, and participants were allowed to interact with the device. To minimize the influence of training on task performance, the usability test was conducted 10 min after the training session [20]. The test environment was arranged to resemble an actual ICU nurse station. Each session was observed in real time using monitoring equipment capable of recording.

Participants conducted 4 use scenarios (consisting of 18 tasks) for 20 min. The scenarios were designed to reflect clinical workflows and included Basic Settings (initial device setup), Cardiac Arrest Prediction (review of patient risk indicators), Patient Review (review of patient data), and Patient Discharge (patient discharge procedures). A risk analysis was conducted to identify tasks where use errors could potentially lead to harm, defined as injury or damage to the health of people, or damage to property or the environment. Tasks that could result in serious outcomes, such as death or permanent injury, were defined as critical tasks. Risk for each task was calculated according to ISO 14971 as the combination of harm probability and severity [22]. All tasks for each scenario are listed in Table 1. After completion of the usability test, participants completed the SUS survey and a satisfaction survey.

### 2.4. Analysis

#### 2.4.1. Usability Test

Participants’ task performance was observed in real time using recording-capable monitoring equipment, and use errors were analyzed based on the recorded data. Task outcomes were categorized as Completed (C), Completed with Issues (CI), or Did Not Complete (NC). C indicates successful task completion without any observed or reported errors; CI refers to cases where errors occurred but were self-corrected (close call) or where difficulties were encountered, yet the task was completed (difficulty); NC includes a task that was not completed, completed incorrectly or required assistance from the moderator [20].

Task success rate was defined as the proportion of participants who achieved either C or CI, relative to the total number of participants. Use error rate was defined as the proportion of participants with NC outcomes. In this study, the goal was to achieve a task success rate of at least 70% and a use error rate below 30%, which was set considering the characteristics of the device and the actual clinical environment.

#### 2.4.2. SUS (System Usability Scale) Survey

The SUS survey is widely used to evaluate the usability of systems across various fields [23]. As shown in Table 2, the SUS consists of 10 items, with odd-numbered items using positive statements and even-numbered items using negative statements [24]. Each item is rated on a 5-point Likert scale. For odd-numbered items, a score of 1 indicates “strongly disagree,” and a score of 5 indicates “strongly agree” [25]. For even-numbered items, a score of 1 indicates “strongly agree,” and a score of 5 indicates “strongly disagree” [25]. To calculate the SUS score, which ranges from 0 to 100, odd-numbered items are scored by subtracting 1 from the user response, and even-numbered items are scored by subtracting the user response from 5. The total sum is then multiplied by 2.5 to determine the SUS score [26]. The SUS score can be interpreted as Worst Imaginable (≤12.5), Poor (12.5–50.8), OK (50.9–71.3), Good (71.4–85.4), Excellent (85.5–90.8), and Best Imaginable (≥90.9) [27].

#### 2.4.3. Satisfaction Survey

A satisfaction survey was conducted after the usability test to evaluate satisfaction with the device. As shown in Table 3, the satisfaction survey consisted of 9 items rated on a 5-point scale. A score of 5 indicated a very positive response, and a score of 1 indicated a very negative response. The satisfaction score was calculated as the average of the participants’ responses across all items.

## 3. Results

### 3.1. Demographic Characteristics

This study was conducted with 22 nurses who had experience using a patient monitor or central monitoring system. The participants had an average age of 38.0 years and an average clinical experience of 13.8 years. Demographic characteristics are shown in Table 4. All participants completed the usability test, SUS survey, and satisfaction survey.

### 3.2. Usability Test

Participants performed four use scenarios consisting of 18 tasks. The overall task success rate was 90%, with a mean success rate of 88% across the three critical tasks, meeting the pre-established goal. Task success rates and use error rates for each scenario are shown in Table 5. The task success rate was 89% for the “Basic Settings” scenario and 94% for “Cardiac Arrest Prediction”. The task success rates were 89% for ‘Basic Settings,’ 94% for ‘Cardiac Arrest Prediction,’ 81% for ‘Patient Review,’ and 100% for ‘Patient Discharge.’

In the ‘Basic Setting’ scenario (Tasks 1–6), all tasks except Task 4 achieved success rates above 90%. Task 4, which involves setting the severity limit, had a success rate of 45%, below the goal of 70%. The low success rate was due to a use error resulting from poor visibility of the setting button.

**Figure 5:**
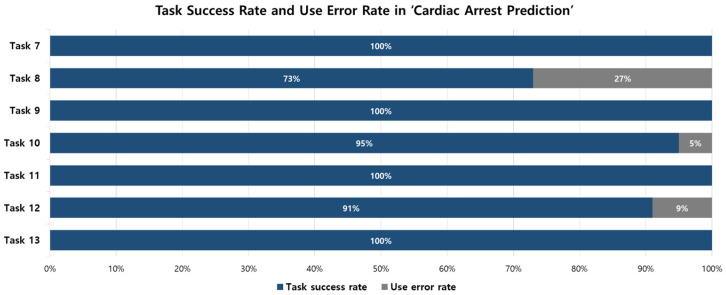
Task success rate and use error rate in ‘Cardiac Arrest Prediction’.

The task success rates for ‘Cardiac Arrest Prediction’ (Tasks 7–13) are shown in Figure 5. Critical tasks that were pre-defined (Tasks 8, 9, and 12) also met the goal. Tasks 8 and 12, which involved confirming the severity of alarms and the patient’s respiration and heart rates, achieved success rates of 73% and 91%. Task 9, which involved pausing the alarm, achieved a 100% success rate.

Tasks 7 and 13 are tasks for checking the patient’s cardiac arrest prediction score, both achieving a success rate of 100%. A difference in the method of displaying alarm severity between InfoWareG (CMS) and the previously used system was identified as a cause of use errors. The previously used system displayed the severity of the alarm using red, yellow, and cyan, whereas InfoWareG (CMS) used red, flashing yellow, and yellow. This difference is expected to lead to a reduction in the user error rate through user training and continued use. Task 9 is a task for pausing the alarm, which achieved a task success rate of 100%. Tasks 10 and 11 are tasks for operating the monitoring screen. Task 10 achieved a task success rate of 95%, and Task 11 achieved 100%.

In the ‘Patient Review’ scenario, Tasks 15 and 16 both achieved 100% success rates. However, Task 14, for checking the patient’s alarm history, and Task 17, for reviewing the cardiac arrest prediction score trends, achieved 68% and 55%, respectively, falling below the 70% goal. The use errors observed in these two tasks were caused by the participants’ inability to locate the required information on the screen, with the root cause being their unfamiliarity with the device.

In the ‘Patient Discharge’ scenario, the task for processing patient discharge achieved a 100% success rate.

### 3.3. SUS (System Usability Scale) Survey

The SUS survey was conducted with 22 ICU nurses who participated in the usability test. The results for each SUS item are shown in Table 6. The SUS score was 67.3 (95% CI 62.6–72.0), which corresponds to an “OK” level.

Odd-numbered items are interpreted as such that higher scores indicate greater user satisfaction. The items related to frequency of use (SUS 1) and ease of learning (SUS 7) received scores of 4.2 and 4.0. These results suggest that users are willing to use the system and can learn how to use it quickly. Even-numbered items are interpreted as such that lower scores indicate greater user satisfaction. The items related to system consistency (SUS 6) and system complexity (SUS 8) received relatively low scores of 2.2 and 2.3. These results indicate that the system is consistent and not overly complex to use.

### 3.4. Satisfaction Survey

A satisfaction survey was conducted with 22 ICU nurses who participated in the usability test. The results for each item of the satisfaction survey are shown in Table 7. The average score of the satisfaction survey was 4.5.

The average satisfaction scores for items 3 and 9, related to the cardiac arrest prediction function, were 4.2 and 4.3. These results indicate that participants were highly satisfied with the system’s ability to predict cardiac arrest. Participants’ feedback indicated that presenting the cardiac arrest prediction score as a numerical value enables intuitive monitoring, thereby increasing its perceived usefulness in clinical practice.

## 4. Discussion

This study performed a summative evaluation of a Central Monitoring System with AI-based Cardiac Arrest Prediction in a simulated environment with 22 ICU nurses to assess whether it can be used safely and effectively. ICUs are designed to provide treatment for critically ill patients, which makes rapid treatment essential, while nurses face high workloads as they must make rapid decisions in critical situations [12]. In addition, frequent alarms and the potential for use errors related to medical devices may affect patient safety, highlighting the need for systematic usability testing before introducing new devices into clinical settings [17,18,19]. The usability test included tasks based on the device workflow, the System Usability Scale (SUS) survey, and a satisfaction survey, and was performed in a simulated environment designed to resemble an ICU to reflect actual clinical environments.

The task success rate in the usability test was 90%, meeting the predefined goal. By scenario, the success rates were 89% for Basic Settings, 94% for Cardiac Arrest Prediction, 81% for Patient Review, and 100% for Patient Discharge. For critical tasks (Tasks 8, 9, and 12), success rates ranged from 73% to 100%. In contrast, Task 4, which involved setting the severity limit, had a success rate of 45%, falling below the goal of 70%. This low success rate was due to poor visibility of the setting button, highlighting a potential use error in a task. Addressing this design issue may improve task success in future device iterations. Additionally, Task 14, for reviewing patient alarm records, and Task 17, for checking the trend of cardiac arrest prediction scores, achieved success rates of 68% and 55%, respectively. These use errors were analyzed to have resulted from poor button visibility, difficulty locating required information on the screen, and participants’ unfamiliarity with the device, as observed during task performance.

The SUS score was 67.3, corresponding to an “OK” level [27], and the satisfaction survey showed an average of 4.5. These results indicate that participants generally found the system acceptable, while use errors arising from differences compared with previously used devices are expected to decrease through user training and continued use.

Although some usability failures were observed during the summative evaluation, their direct impact on patient safety could not be assessed within the simulated environment. Since this study was conducted in a simulated environment, further research is needed to evaluate the system’s effectiveness and user experience in a real clinical environment. Future studies should assess the actual incidence of cardiac arrest events, the time required to initiate interventions, and the extent to which the cardiac arrest prediction score contributes to clinical decision making and timely response. Such research would help clarify how effectively the system supports early detection of cardiac arrest risk and the timely initiation of interventions in a real clinical environment.

## 5. Conclusions

This study evaluated the usability of a Central Monitoring System with Cardiac Arrest Prediction through a usability test, SUS survey, and satisfaction survey. Some tasks showed lower performance due to design issues such as poor button visibility and unfamiliarity with the device, highlighting potential areas for design improvement. As the evaluation was performed in a simulated setting, further studies in real clinical environments are needed to assess effectiveness, user experience, and the system’s contribution to timely detection of cardiac arrest events.

## Figures and Tables

**Table 1:** Use scenario.

Task Number	Task Description	Critical Task
Basic Settings		
Task 1	Enter information for predicting the patient’s critical condition and confirm its application to the PM.	
Task 2	Change the NIBP automatic measurement interval to 60 min and measure NIBP.	
Task 3	Change the HR Alarm Limit to 80/60 and confirm its application to the PM.	
Task 4	Set the primary alarm threshold to 40 and the secondary alarm threshold to 70 for cardiac arrest alarms.	
Task 5	Temporarily silence the alarm for 2 min.	
Task 6	Set the HR Alarm Limit to 100/80 to prevent unnecessary alarms after the temporary silence period ends.	
Cardiac Arrest Prediction		
Task 7	Check the patient’s cardiac arrest prediction score.	
Task 8	Check the severity of the alarm that occurred and the patient’s RR.	✔
Task 9	After confirming the alarm status, temporarily silence the alarm.	✔
Task 10	Change the monitoring screen to a 6-bed view for enhanced patient monitoring.	
Task 11	Change the monitoring screen to a 4-bed view to focus on patients with higher cardiac arrest prediction scores.	
Task 12	Check the severity of the occurred alarm and the patient’s HR.	✔
Task 13	Check the patient’s cardiac arrest prediction score.	
Patient Review		
Task 14	Open the patient’s ECG waveform review window to check the history of ECG-related alarms.	
Task 15	Zoom in on the waveform in the area where the Asystole alarm occurred.	
Task 16	Set the HR to be displayed.	
Task 17	Review the trend data of the patient’s cardiac arrest prediction score.	
Patient Discharge		
Task 18	Discharge the patient from Bed 1.	

**Table 2:** System Usability Scale (SUS) items.

No	System Usability Scale (SUS) Items
1	I think that I would like to use this system frequently.
2	I found the system unnecessarily complex.
3	I thought the system was easy to use.
4	I think that I would need the support of a technical person to be able to use this system.
5	I found the various functions in this system were well integrated.
6	I thought there was too much inconsistency in this system.
7	I would imagine that most people would learn to use this system very quickly.
8	I found the system very cumbersome to use.
9	I felt very confident using the system.
10	I needed to learn a lot of things before I could get going with this system.

**Table 3:** Satisfaction survey items.

No	Satisfaction Survey Items
1	Do you think the function of remotely changing the information entered into the PM through CMS is useful?
2	Do you think the function of remotely changing and measuring the NIBP auto-measurement interval of PM is useful?
3	Do you think the function of predicting cardiac arrest and displaying the score based on artificial intelligence is useful?
4	Do you think the function of monitoring multiple patients simultaneously is useful?
5	Do you think the function of changing the alarm limits is useful?
6	Do you think the function of pausing the alarm when it occurs is useful?
7	Do you think the function of categorizing alarms by severity (High, Medium, Low) is useful?
8	Do you think the function of zooming on the area where ECG-related alarms occur for confirmation is useful?
9	Do you think the function of checking the trend of the cardiac arrest prediction score is useful?

**Table 4:** Demographic characteristics and experience of usability test participants.

Variable		Users
Gender	Male	3
	Female	19
Age	20–29 years	6
	30–39 years	5
	40–49 years	10
	50–59 years	1
Department of participant variable	Intensive Care Unit (ICU)	22
Work experience	Less than 3 years	2
	More than 3 years, less than 5 years	3
	More than 5 years, less than 10 years	6
	More than 10 years	11
User experience with similar devices	Less than 3 years	2
	More than 3 years, less than 5 years	3
	More than 5 years, less than 10 years	6
	More than 10 years	11

**Table 5:** Task success rate and user error rate by use scenarios.

Use Scenario	Task Success Rate	Use Error Rate
Basic Settings	89%	11%
Cardiac Arrest Prediction	94%	6%
Patient Review	81%	19%
Patient Discharge	100%	0%

**Table 6:** SUS survey results.

No	System Usability Scale (SUS) Items	Mean	SD
1	I think that I would like to use this system frequently.	4.2	0.6
2	I found the system unnecessarily complex.	2.9	1.0
3	I thought the system was easy to use.	3.8	0.7
4	I think that I would need the support of a technical person to be able to use this system.	3.0	1.0
5	I found the various functions in this system were well integrated.	4.0	0.7
6	I thought there was too much inconsistency in this system.	2.2	1.0
7	I would imagine that most people would learn to use this system very quickly.	4.0	0.6
8	I found the system very cumbersome to use.	2.3	0.7
9	I felt very confident using the system.	3.8	0.8
10	I needed to learn a lot of things before I could get going with this system.	2.5	0.9
System Usability Scale (SUS) Score		67.3	11.2

**Table 7:** Satisfaction survey results.

No	Satisfaction Survey Items	Mean	SD
1	Do you think the function of remotely changing the information entered into the PM through CMS is useful?	4.5	0.6
2	Do you think the function of remotely changing and measuring the NIBP auto-measurement interval of PM is useful?	4.6	0.6
3	Do you think the function of predicting cardiac arrest and displaying the score based on artificial intelligence is useful?	4.2	0.8
4	Do you think the function of monitoring multiple patients simultaneously is useful?	4.7	0.5
5	Do you think the function of changing the alarm limits is useful?	4.7	0.5
6	Do you think the function of pausing the alarm when it occurs is useful?	4.6	0.5
7	Do you think the function of categorizing alarms by severity (High, Medium, Low) is useful?	4.1	1.0
8	Do you think the function of zooming on the area where ECG-related alarms occur for confirmation is useful?	4.4	0.7
9	Do you think the function of checking the trend of the cardiac arrest prediction score is useful?	4.3	0.7

## Data Availability

Data are available upon demand.

## Abbreviations

The following abbreviations are used in this manuscript:

ICU	intensive care unit
CMS	Central Monitoring System
ECG	electrocardiogram
CO_2_	carbon dioxide
SpO_2_	transcutaneous oxygen saturation
HR	heart rate
NIBP	non-invasive blood pressure
PR	pulse rate
RR	respiration rate
IRB	Institutional Review Board
SUS	System Usability Scale

## References

[1] Lee H., Yang H.L., Ryu H.G., Jung C.W., Cho Y.J., Yoon S.B., Yoon H.K., Lee H.C. (2023). Real-Time Machine Learning Model to Predict in-Hospital Cardiac Arrest Using Heart Rate Variability in ICU. npj Digit. Med..

[2] Armstrong R.A., Kane C., Oglesby F., Barnard K., Soar J., Thomas M. (2019). The Incidence of Cardiac Arrest in the Intensive Care Unit: A Systematic Review and Meta-Analysis. J. Intensive Care Soc..

[3] Kwon J.M., Kim K.H., Jeon K.H., Lee S.Y., Park J., Oh B.H. (2020). Artificial Intelligence Algorithm for Predicting Cardiac Arrest Using Electrocardiography. Scand. J. Trauma Resusc. Emerg. Med..

[4] Hillman K.M., Bristow P.J., Chey T., Daffurn K., Jacques T., Norman S.L., Bishop G.F., Simmons G. (2002). Duration of Life-Threatening Antecedents Prior to Intensive Care Admission. Intensive Care Med..

[5] Elvekjaer M., Aasvang E.K., Olsen R.M., Sørensen H.B.D., Porsbjerg C.M., Jensen J.U., Haahr-Raunkjær C., Meyhoff C.S. (2020). Physiological Abnormalities in Patients Admitted with Acute Exacerbation of COPD: An Observational Study with Continuous Monitoring. J. Clin. Monit. Comput..

[6] Lee A., Bishop G., Hillman K.M., Daffurn K. (1995). The Medical Emergency Team. Anaesth. Intensive Care.

[7] Smith G.B., Osgood V.M., Crane S. (2002). ALERT—A Multiprofessional Training Course in the Care of the Acutely Ill Adult Patient. Resuscitation.

[8] Shin Y., Cho K.-j., Chang M., Youk H., Kim Y.J., Park J.Y., Yoo D. (2024). The Development and Validation of a Novel Deep-Learning Algorithm to Predict in-Hospital Cardiac Arrest in ED-ICU (Emergency Department-Based Intensive Care Units): A Single Center Retrospective Cohort Study. Signa Vitae.

[9] Kim Y.K., Seo W.D., Lee S.J., Koo J.H., Kim G.C., Song H.S., Lee M. (2024). Early Prediction of Cardiac Arrest in the Intensive Care Unit Using Explainable Machine Learning: Retrospective Study. J. Med. Internet Res..

[10] Li Y., Ye W., Yang K., Zhang S., He X., Jin X., Wang C., Sun Z., Liu M. (2022). Prediction of Cardiac Arrest in Critically Ill Patients Based on Bedside Vital Signs Monitoring. Comput. Methods Programs Biomed..

[11] Giebel G.D., Raszke P., Nowak H., Palmowski L., Adamzik M., Heinz P., Tokic M., Timmesfeld N., Brunkhorst F.M., Wasem J. (2025). Improving AI-Based Clinical Decision Support Systems and Their Integration into Care from the Perspective of Experts: Interview Study Among Different Stakeholders. JMIR Med. Inform..

[12] Nasirizad Moghadam K., Chehrzad M.M., Reza Masouleh S., Maleki M., Mardani A., Atharyan S., Harding C. (2021). Nursing Physical Workload and Mental Workload in Intensive Care Units: Are They Related?. Nurs. Open.

[13] Choi H., Kim Y., Jang W. (2025). Enhancing the Usability of Patient Monitoring Devices in Intensive Care Units: Usability Engineering Processes for Early Warning System (EWS) Evaluation and Design. J. Clin. Med..

[14] Andrade E., Quinlan L., Harte R., Byrne D., Fallon E., Kelly M., Casey S., Kirrane F., O’Connor P., O’Hora D. (2020). Novel Interface Designs for Patient Monitoring Applications in Critical Care Medicine: Human Factors Review. JMIR Hum. Factors.

[15] Kim Y., Son J., Jang W. (2023). Usability Study on Patient Monitoring Systems: An Evaluation of a User Interface Based on User Experience and Preference. Med. Sci. Monit..

[16] Mohammadi M., Mazloumi A., Kazemi Z., Zeraati H. (2015). Evaluation of Mental Workload among ICU Ward’s Nurses. Health Promot. Perspect..

[17] Dee S.A., Tucciarone J., Plotkin G., Mallilo C. (2022). Determining the Impact of an Alarm Management Program on Alarm Fatigue among ICU and Telemetry RNs: An Evidence Based Research Project. SAGE Open Nurs..

[18] Daniels J., Fels S., Kushniruk A., Lim J., Ansermino J.M. (2007). A Framework for Evaluating Usability of Clinical Monitoring Technology. J. Clin. Monit. Comput..

[19] Choi H., Jang W. (2024). User Experience Study of the Patient Monitoring Systems Based on Usability Testing and Eye Tracking. Healthcare.

[20] Privitera M.B. (2019). Applied Human Factors in Medical Device Design.

[21] (2013). Human Factors Engineering-Design of Medical Devices.

[22] (2019). Medical Devices—Application of Risk Management to Medical Devices.

[23] Sevilla-Gonzalez M.D.R., Moreno Loaeza L., Lazaro-Carrera L.S., Bourguet Ramirez B., Vázquez Rodríguez A., Peralta-Pedrero M.L., Almeda-Valdes P. (2020). Spanish Version of the System Usability Scale for the Assessment of Electronic Tools: Development and Validation. JMIR Hum. Factors.

[24] Mohamad Marzuki M.F., Yaacob N.A., Yaacob N.M. (2018). Translation, Cross-Cultural Adaptation, and Validation of the Malay Version of the System Usability Scale Questionnaire for the Assessment of Mobile Apps. JMIR Hum. Factors.

[25] Østervang C., Jensen C.M., Coyne E., Dieperink K.B., Lassen A. (2024). Usability and Evaluation of a Health Information System in the Emergency Department: Mixed Methods Study. JMIR Hum. Factors.

[26] Deshmukh A.M., Chalmeta R. (2024). Validation of System Usability Scale as a Usability Metric to Evaluate Voice User Interfaces. PeerJ Comput. Sci..

[27] Bangor A., Kortum P., Miller J. (2009). Determining What Individual SUS Scores Mean: Adding an Adjective Rating Scale. J. Usability Stud..

